# Limbal Stem Cell Deficiency: Current Treatment Options and Emerging Therapies

**DOI:** 10.1155/2016/9798374

**Published:** 2015-12-14

**Authors:** Michel Haagdorens, Sara Ilse Van Acker, Veerle Van Gerwen, Sorcha Ní Dhubhghaill, Carina Koppen, Marie-José Tassignon, Nadia Zakaria

**Affiliations:** ^1^Faculty of Medicine and Health Sciences, Department of Ophthalmology, Visual Optics and Visual Rehabilitation, University of Antwerp, Campus Drie Eiken, T building, T4-Ophthalmology, Universiteitsplein 1, 2610 Antwerp, Belgium; ^2^Department of Ophthalmology, Antwerp University Hospital, Dienst Oogheelkunde, Wilrijkstraat 10, 2650 Edegem, Belgium; ^3^Research Foundation-Flanders, Egmontstraat 5, 1000 Brussels, Belgium; ^4^Center for Cell Therapy and Regenerative Medicine, Antwerp University Hospital, CCRG-Oogheelkunde, Wilrijkstraat 10, 2650 Edegem, Belgium

## Abstract

Severe ocular surface disease can result in limbal stem cell deficiency (LSCD), a condition leading to decreased visual acuity, photophobia, and ocular pain. To restore the ocular surface in advanced stem cell deficient corneas, an autologous or allogenic limbal stem cell transplantation is performed. In recent years, the risk of secondary LSCD due to removal of large limbal grafts has been significantly reduced by the optimization of cultivated limbal epithelial transplantation (CLET). Despite the great successes of CLET, there still is room for improvement as overall success rate is 70% and visual acuity often remains suboptimal after successful transplantation. Simple limbal epithelial transplantation reports higher success rates but has not been performed in as many patients yet. This review focuses on limbal epithelial stem cells and the pathophysiology of LSCD. State-of-the-art therapeutic management of LSCD is described, and new and evolving techniques in ocular surface regeneration are being discussed, in particular, advantages and disadvantages of alternative cell scaffolds and cell sources for cell based ocular surface reconstruction.

## 1. Introduction

**Figure 1:**
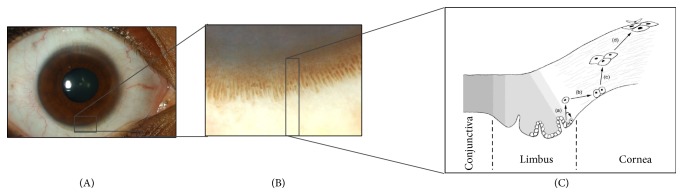
(A) Overview of the anterior surface of the human eye, in which the sclera (with overlying conjunctiva) and cornea can easily be discriminated. (B) The limbus is highly pigmented in some individuals, and allows clear visualization of the limbal palisades of Vogt. The cornea (and underlying dark iris) is pictured above, and conjunctiva (and underlying sclera) below. (C) Diagram of a cross section through the conjunctival, limbal and corneal epithelium. Limbal progenitor cells (a) differentiate into transient amplifying cells (b), post-mitotic cells (c) and finally terminally differentiated cells (d). Movement of cells in X, Y, Z direction is presented by proliferation of stem cells(a), differentiation and centripetal migration (b, c), and desquamation (d) respectively.

Located at the anterior segment of the eye, the cornea is highly organised transparent tissue consisting of multiple cellular and noncellular layers [1]. The corneal epithelium covers the corneal surface and plays a major role in protection and transparency [2, 3]. Epithelial cells are shed regularly and replaced by stem cell sources located at the limbus, a rim of tissue located at the junction of the cornea and sclera (Figures 1(A) and 1(B)). The limbal epithelial stem cells (LESCs) reside in specific regions at the limbus known as the limbal stem cell niches [4]. Damage to the stem cells or disruption of the niches may lead to Limbal Stem Cell Deficiency (LSCD). In the absence of a healthy corneal epithelium, the conjunctiva proliferates over the cornea resulting in opacification and vascularization, which in turn may lead to reduced vision, pain, and photophobia [5, 6]. LSCD can be caused by a wide variety of primary and secondary causes (Table 1) but is most frequently seen associated with severe chemical or thermal burns.

Diagnosis of LSCD is often on the bases of history and clinical findings, which include loss of limbal anatomy, corneal conjunctivalization, persistent epithelial defects, and scar formation [7, 8]. In partial LSCD clinical signs are present but limited to specific regions, which may be quantified by the number of limbal clock hours involved. The diagnosis is confirmed by impression cytology [9], illustrating the presence of goblet cells, increased cytokeratin 19 (CK19) expression, and reduced CK3/12 expression [10]. More recently CK7, mucin1, and mucin5AC have been reported as more specific than CK19 for diagnostic purposes [11–14].

*In vivo* confocal microscopy (IVCM) and anterior optical coherence tomography (OCT) are promising techniques that may assist in diagnosing and quantifying LSCD and guiding therapeutic management. IVCM provides high-resolution images of anatomical structures at the cellular level [15, 16]. A number of practical factors limit its use; firstly there is no consensus on the definitive morphological appearance of LESCs, surrounding niche cells or goblet cells on IVCM [17, 18]. Secondly, in the presence of a hazy cornea, the technique is less effective in defining structures due to high degree of backscatter, and finally it requires the prolonged cooperation of the patient [19]. Anterior OCT, and in particular Fourier Domain OCT (FD-OCT), is a more rapid and convenient method of imaging limbal, scleral, and conjunctival structures, though, with significantly lower resolution than IVCM [20]. 3D guided reconstructions of the limbus can be made and may assist guided limbal biopsy [20]. Furthermore, FD-OCT can be applied in imaging hazy corneas and facilitates intraoperative dissection of fibrovascular pannus.

## 2. Treatment of LSCD

Therapeutic options for LSCD range from conservative to invasive and depend on the severity of the pathology (Table 2). Conservative therapeutic options include supportive management, corneal scraping, and amniotic membrane patching. In these cases, recovery depends on the presence of some remaining LESCs that can be rehabilitated to restore the epithelium. If there are no remaining stem cell reserves, the cornea must be reseeded with new LESCs [7, 21]. Over the past 18 years, optimizing reseeding techniques has been a major focus of corneal tissue engineering. The earliest techniques required large sections of donor tissue either from the patient's fellow eye (autograft) or from a healthy donor or cadaver (allograft). Taking such large biopsies places the donor eye at risk of developing LSCD. In 1997, Pellegrini et al. reported the first application of* ex vivo* expansion of a very small stem cell biopsy in the treatment of LSCD [22]. The* ex vivo* technique significantly reduced the risk to the donor eye. Since the original report, numerous clinical trials have reported outcomes of tissue engineered corneal surface reconstruction [22–60]. This review will focus on the nature of LESCs and the evolution and optimization of cultivated limbal epithelial stem cell transplantation (CLET) as well as possible future directions.

## 3. Limbal Epithelial Stem Cell Niches and Markers

A stem cell niche is the unique microenvironment that surrounds stem cells and modulates their function and fate through internal and external factors. LESCs reside in a such well-protected microenvironment, the limbal stem cell niche. The niches are protected from UV-radiation by (i) melanocytes that reside in the basal layers of the limbal epithelium and (ii) the upper and lower eyelid that offer cover to the superior and inferior limbus [8, 61, 62]. The niche's undulated basement membrane protects LESCs from shear force, whereas limbal stromal blood vessels and mesenchymal cells supply it with oxygen, cytokines, growth factors (e.g., the keratinocyte growth factor), and other nutrients [16, 63–65]. The niche also regulates the LESC cell cycle to keep them in an undifferentiated resting state [16, 66]. Proliferation of a LESC gives rise to two daughter cells, where one remains an oligopotent LESC and the other differentiates into a transient amplifying cell (TAC). After a high but limited number of mitoses, TACs differentiate into “postmitotic cells” and subsequently “terminally differentiated cells” [67–69] (Figure 1(C)). During this differentiation process, cells migrate centripetally from the niche to the corneal surface [4] according to the *XYZ*-hypothesis [70], that is, proliferation of basal epithelial cells (*x*), differentiation and centripetal migration (*y*), and isolation/desquamation (*z*).

**Figure 2:**
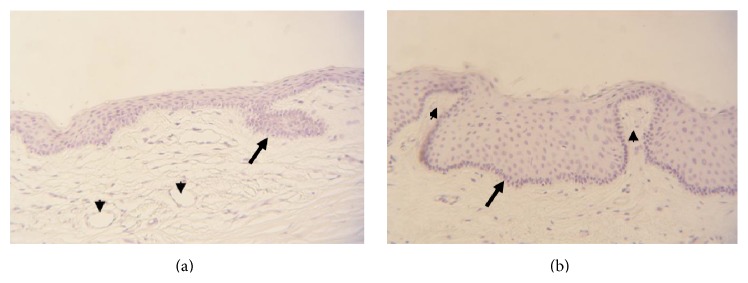
Haematoxylin staining of cross section through normal limbal region. Arrow in (a) indicates a LESC containing limbal epithelial crypt; arrowheads indicate blood vessels. Arrow in (b) indicates a limbal crypt, flanked by two focal stromal projections (arrowhead).

Recently, Molvaer et al. localized and described the three different limbal stem cell niches, (i) the limbal epithelial crypts (LECs), (ii) the limbal crypts (LCs), and (iii) the focal stromal projections (FSPs) (Figure 2) [98]. LECs were first described in 2005 as projections extending from the undersurface of the limbal epithelium into the stroma. These projections extend radially into the conjunctival stroma parallel to the palisade or circumferentially along the limbus at right angles to the palisade (Figure 2(a)) [99]. In 2007, LCs and FSPs were described as additional stem cell niches. LCs are projections of the limbal epithelium into the stroma, which are laterally enclosed by the palisades of Vogt [16]. The defined area corresponds in part to the previously described interpalisades (Figure 2(b)). FSPs are finger-shaped projections of stroma containing a central blood vessel, which extend upward into the limbal epithelium [16]. More recently, a further subdivision was made between basal and superficial LCs, the former containing LESCs with melanocytes, the latter containing TACs [100]. It has been proven that all three limbal stem cell niches are mainly present at the superior, and to lesser extent, the inferior limbus. There is no consensus, however, about the exact number and location of niches in the limbus [98].

Stemness and differentiation of LESCs have been investigated through the analysis of various cell markers. Though no specific marker for LESCs has been identified [101, 102], ABCG2 (also known as BRCP1) [103], p63*α* [104], and ΔNp63 [105] isoforms are the leading markers used in putative LESC identification. Additional stem cell markers have been described, with integrins *α*v*β*3/5 and the ABCB5 gene most recently [106, 107]. Ordonez et al. identified integrin *α*v*β*3/5 in less than 4% of cells present in the limbal epithelial niche. However, these cells had phenotypic and functional LESC properties [106].

## 4. Cultured Limbal Epithelial Stem Cell Transplantation 

As a technique, cultured limbal epithelial stem cells transplantation (CLET) is in its infancy. The overall success rate is estimated to be 76% [21], though direct comparison of clinical trials is difficult due to the wide diversity of pathologies treated, culture protocols, surgical approach, and subjective and objective outcome parameters. When recently published clinical reports are taken into account, success rate decreases slightly to 70%. Details on culture methods and clinical results of published reports are described (Table 3). No significant differences were found in the clinical outcomes based on initial cause of LSCD, source of donor tissue (autologous or allogenic), or culture technique (explants or suspension) [21, 93]. Some culture protocols require the use of lethally irradiated or Mitomycin C-treated 3T3 feeder cells, either in direct contact or in coculture with the LESCs [25, 29, 31, 35, 37, 41–45, 47, 50–52, 55, 58–60]. The feeder layers are involved in promoting niche regulation and stemness of cultivated cells. Though no adverse reactions have been reported in the use of 3T3 feeder layers in large case series [108, 109], avoiding xenogenic material may help reduce the risk of animal-derived infection and graft rejection. The search for alternatives to bovine and other animal products in cultivation protocols, for example, fetal bovine serum and animal-derived growth factors, has led to recent clinical studies cultivating LESCs under nonxenogenic conditions [52, 54, 55, 57–59]. Other advances in the field that may also translate to a higher success rate in future trials include feeder layers of human fibroblasts or Mesenchymal Stem Cells (MSCs) [110–114], standardized GMP (Good Manufacturing Practice) protocols [115] for HAM preparation and* ex vivo* culture, use of autologous serum drops postoperatively, and minimal manipulation of the graft during transplantation [116–118].

In 2012, simple limbal epithelial transplantation (SLET) was described as a novel surgical technique for the treatment of unilateral LSCD [94]. During SLET surgery, a small strip of donor limbal tissue (e.g., 2 × 2 mm) is divided into several smaller pieces, which are then distributed evenly over a HAM placed on the cornea [94]. The surgery obviates the need for a culture protocol entirely. Although each clinical study reported a success rate of 100% in a small case series (Table 4) [94–97, 119, 120], the long-term effectiveness of the technique is yet to be proven.

## 5. Alternative Cell Carriers 

In clinical trials, HAM is the most commonly used cell carrier for ocular surface reconstruction [23–27, 29–33, 35–42, 44, 45, 47, 48, 50–60]. However, there are risks associated with the use of HAM including possible transfer of infectious agents, variable tissue quality, and limited transparency, which is why alternative seeding scaffolds have been proposed [42, 121].

### 5.1. Modified HAM

Chemical crosslinking of HAM may enhance mechanical and thermal stability, optical transparency, and resistance to collagenase digestion [122–126]. The crosslinking agents that have been investigated are Glutaraldehyde, (L-Lysine-modulated) Carbodiimide, and Al_2_(SO_4_)_3_ [122–126].* In vitro* experiments showed that Glutaraldehyde conferred a higher degree of cytotoxicity than Carbodiimide [123], whereas the addition of L-lysine to the Carbodiimide crosslinking enhanced mechanical and thermal strength, the ability to support LESCs, and resistance to enzymatic digestion, though higher concentrations could compromise transparency and biocompatibility [126].

### 5.2. Collagen

Collagen is the main extracellular matrix protein of the cornea and has been widely investigated in the development of biomimetic carrier materials. It is naturally biocompatible and relatively inexpensive to isolate [127, 128]. LESCs can be successfully cultivated on collagen carriers, while maintaining normal phenotype and achieving multilayered stratification when transplanted* in vivo *[127, 129, 130]. Cell attachment and proliferation can be further improved, by coating scaffolds with extracellular matrix proteins (e.g., laminin, type IV collagen, and fibronectin) or derivative adhesion peptides (e.g., YIGSR, IKVAV, and RGC) [131–137]. Most experimental studies have been performed using animal-derived collagen (e.g., porcine collagen type I, rat tail collagen type I, bovine dermal collagen, and fish scale) [127, 138–144]. This collagen may transmit diseases or induce immune reactions, and therefore the more expensive recombinant human collagen (RHC) type I and type III are being investigated further for clinical translation [145–151]. Despite the advantages associated with their use, collagen hydrogels are inherently weak due to the high water content [152]. Several methods have been proposed to improve the mechanical properties of collagen hydrogels.

#### 5.2.1. Chemically Crosslinked Collagen

Griffith et al. have reported the construction of biosynthetic collagen scaffolds consisting of concentrated type I and type III RHC solutions, crosslinked with 1-ethyl-3-(3-dimethyl aminopropyl) Carbodiimide (EDC) and N-hydroxysuccinimide (NHS) [153–155]. When LESCs were cultivated* in vitro *on the optically transparent constructs, a stratified epithelium formed and covered the surface within three weeks. The constructs were sufficiently robust to provide adequate mechanical stability and elasticity for surgical manipulation. Type III collagen hydrogels tended to be mechanically superior.* In vivo* verification and validation showed that the acellular scaffolds stayed optically clear and promoted regeneration of corneal cells, nerves, and tear film, without the need for long-term immunosuppression [149]. However, the mechanical properties of the constructs were significantly lower than human corneas and the long-term stability still needs to be ascertained.

To improve the mechanical properties of the constructs, Griffith et al. have investigated reinforced membranes fabricated from EDC/NHS crosslinked type III RHC and PEG-diacrylate crosslinked 2-methacryloyloxyethyl phosphorylcholine (MPC) [151, 156–158]. These hydrogels showed increased mechanical strength and stability against enzymatic digestion and UV degradation and promoted corneal cell and nerve regeneration while optical properties were comparable to a normal cornea [156]. Cell-free RHC-MPC implants have been grafted in 7 eyes, in which patients showed stable epithelia 12 months postoperatively and the best corrected vision improved by 1-2 lines [151, 158]. Another form of collagen hydrogel, genipin-crosslinked chitosan-collagen and PEG-Carbodiimide chitosan-collagen hydrogel, has also been examined for ocular surface reconstruction [139, 159].* In vitro *experiments with these constructs show maintenance of regular stratified multilayered epithelium [159], while initial animal testing shows good biocompatibility [139]. Use in human corneal regeneration has not yet been reported.

#### 5.2.2. Plastic Compression Collagen

In 2010, Mi et al. improved the mechanical strength of collagen hydrogels by compressing and blotting the constructs between paper sheets and a nylon mesh thereby reducing the water content of the gels [160]. LESCs cultivated on this construct displayed a smooth and homogenous morphology, whereas cells cultured on conventional hydrogels were distributed more heterogeneously. Subsequent studies confirmed that plastically compressed collagen gels are optically transparent and easy to handle, had improved mechanical strength, and support LESC adhesion, proliferation, and stratification [160–163]. Mechanical strength could further be improved by photochemical crosslinking [164]. Kits that enable the production of 3D plastic compressed cultures have recently become commercially available (RAFT, TAP Biosystems, Hertfordshire, UK).

### 5.3. Fibrin

Fibrin is the biodegradable product formed during coagulation. Fibrin membranes can be fabricated by combining fibrinogen and thrombin, both harvested from human plasma. Fibrin derivates have been used extensively in ophthalmology, typically as a glues or membranes [165–168].

Four clinical studies have reported the use of fibrin as a substrate in CLET surgery [28, 46, 48, 49]. In animal studies, fibrin gels were found to degrade completely after 3 days [169]. After gel degradation, the transplanted cells adhered directly to the host corneal stroma. In early 2015, Holoclar (Chiesi, Italy) has been conditionally approved to be released in Italy as the first commercially available stem cell therapy for LSCD treatment. Existing data on Holoclar have been obtained by retrospective patient follow-up, and annual renewal of approval will be guided by results of a current multicenter, prospective phase IV clinical trial. Nevertheless, practical use of this fibrin-based Advanced Therapeutic Medicinal Product (ATMP) is limited to autologous stem cell transplantation in unilateral cases after chemical or thermal burn. Notably, the technique still utilizes lethally irradiated murine 3T3-J2 fibroblast feeder cells and bovine serum during graft generation, which brings into question the safety of the xeno-based cell product [49].

### 5.4. Siloxane Hydrogel Contact Lenses

In the initial CLET clinical trial by Lu et al., a 3T3 cocultured human epithelial sheet was mounted on a soft contact lens, prior to transplantation as a carrier [170]. In a subsequent study by Di Girolamo et al., the LESCs were cultivated directly on the contact lens [171]. Gore et al. investigated cultivation of LESCs on contact lenses that were coated with a 3T3 feeder layer [172]. In this study,* in vitro *cultivated LESCs formed a multilayered corneal epithelium, while some basal cells maintained their stemness. Plasma polymer-coated contact lenses also promoted* in vitro* LESC adhesion and proliferation [173]. Transplantation of these LESCs in a LSCD rabbit model gave rise to patches of stratified epithelium; however, recipient corneas showed only partial reconstruction, possibly due to short-term follow-up (26 days).

### 5.5. Poly(*ε*-caprolactone)

Poly(*ε*-caprolactone) is a highly flexible and strong material that has already been used as a scaffold for skin, bone, and MSC applications. The biocompatibility and optical transparency of poly(*ε*-caprolactone) may be improved by electrospinning and surface modification, and such modified sheets can support LESC cultivation [174]. The* in vivo* use of the material has not yet been reported.

### 5.6. Chitosan-Gelatin

Chitosan is a stiff crystalline polysaccharide that is extracted from chitin from arthropod exoskeletons. Membranes of pure chitosan are too stiff for ocular purposes but the addition of gelatine and crosslinkers can improve the material handling [175]. Chitosan-gelatine membranes have extensively been investigated for regeneration of bone, cartilage, and skin [176–178]. Chitosan-gelatin membranes with a 20 : 80 ratio supported the growth of LESCs that expressed CK3/12, CK15, and ABCG2 [179]. Again, the* in vivo* use of this material has not been reported.

### 5.7. Silk Fibroin

Silk fibroin (SF), obtained from* Bombyx mori* (domesticated silkworm), can be processed into thin transparent membranes. It is nonimmunogenic, degradable, mechanically strong, and optically transparent and has been used as suture material and in bone and cartilage regeneration [180–182]. Cultivation of LESCs on nonporous SF films gives rise to a stratified corneal-like epithelium [183–187]. Porous SF membranes can be developed by mixing SF and poly(ethylene glycol) (PEG) and have supported LESC growth [183] although results have varied [186]. It may be possible to coculture MSCs within pores to recreate the stromal microenvironment [186]. SF may also be combined with chitosan (SF-CS) and the constructed scaffolds have been investigated with some success [188, 189]. LESCs that were seeded on such lamellar corneas were comparable to native tissue, as outgrown cells had physiological morphology and high levels of CK3/12 expression [189]. Furthermore, biocompatibility of SF and SF-CS films has been observed in rabbit corneas for up to six months [183, 188]. However, membranes constructed from SF derived from* Antheraea pernyi *(wild silkworm) proved to be more prone to becoming opaque, displayed lower permeability, and were more brittle than conventional nonporous SF films [187].

### 5.8. Human Anterior Lens Capsule

The Human Anterior Lens Capsule (HaLC) is a dense membrane consisting of Collagen IV, laminin, and heparin sulphate proteoglycans. HaLC is characterized by a gradually increasing thickness (±0.35 *µ*m per year) and simultaneous loss of mechanical strength (±1% each year) [190, 191]. LESCs have been successfully cultivated on HaLCs, with* in vitro* viability of >95%; cell density and cell morphology were similar to LESCs cultivated on plastic [192]. LESCs, cultured under nonxenogenic conditions maintained their oligopotency, while some cells showed directional differentiation into corneal epithelium [193]. This promising alternative scaffold needs further* in vivo* verification. Concern has been raised, however, that the diameter of extracted HaLC may not be large enough for corneal treatments [192].

### 5.9. Keratin

Reichl et al. succeeded in fabricating a transparent membrane from keratin extracted from human hair [194]. LESC behavior on the films was similar to that on HAM and was not affected by prior plasma treatment sterilization of the material [195]. Unfortunately, suturing is impaired by a high rate of suture tear-out [195].

### 5.10. Poly(lactide-co-glycolide)

Poly(lactide-co-glycolide) (PLGA) is an FDA-approved, biodegradable, and noncytotoxic material that has been used in products such as dissolvable sutures [196]. Transparent electrospun PLGA scaffolds are easy to handle, store, and suture [197]; however when LESCs were cultivated on these carriers, the scaffolds began to disintegrate* in vitro* and were fragile to handle. Additional research has shown that PLGA can be chemically altered to achieve predictable and slower breakdown, both* in vitro* and* in vivo *[198, 199]. Disintegration was now evident by two weeks after initiation of LESC cultivation, with complete breakdown occurring by six weeks* in vitro* [199].

### 5.11. Polymethacrylate

Polymethacrylate has been used in ophthalmology to produce rigid intraocular lenses and contact lenses. It can be fabricated into transparent biocompatible hydrogels, which can support LESC proliferation [200, 201]. Augmenting the polymethacrylate with 1,4-diaminobutane has been shown to improve LESC adherence and proliferation [202].

### 5.12. Hydroxyethylmethacrylate

Hydroxyethylmethacrylate and poly-2-hydroxyethylmethacrylate have been used to manufacture soft contact lenses, the Chirila Kpro and the AlphaCor (Addition Technology Inc., Des Plaines, IL) [203, 204]. One study has investigated hydroxyethylmethacrylate in ocular surface reconstruction and concluded that LESCs and fibroblasts could adhere and proliferate to hydroxyethylmethacrylate hydrogels that were surface modified with type I collagen and arginine-glycine-aspartic acid ligand [205].

### 5.13. Poly(ethylene glycol)

PEG is a biocompatible polymer used in pharmaceutical products (e.g., capsules, tablet binders, ointments, and slow release medications). Transparent hydrogels based on PEG-diacrylate and PEG-diacrylamide have been used* in vivo* and showed favourable results for the latter as PEG-diacrylate implants showed inflammation, corneal haze, and corneal ulceration. Rabbits with PEG-diacrylamide implants, on the other hand, remained healthy and had clear corneas and noninflamed eyes for up to 6 months after transplantation [206, 207].* In vitro *experiments showed that photolithographical surface coating with collagen type I was necessary to allow LESC adhesion and proliferation [208]. PEG-diacrylate and PEG-diacrylamide hydrogels were intended for full thickness corneal regeneration; however, thinner gels intended for anterior corneal regeneration are yet to be investigated. PEG has also been combined with chitosan and silk fibroin to make even stronger and more transparent biomaterials [209].

### 5.14. Platelet Poor Plasma

Platelet-Poor Plasma (PPP) is blood plasma with very low numbers of thrombocytes (<10 × 10^3^/*μ*L), which are removed by centrifugation. Biodegradable, transparent PPP membranes can be manufactured to function as a seeding scaffold in autologous and allogenic CLET. LESC allografts mounted on autologous PPP sheets in LSCD rabbits improved corneal transparency and resulted in a multilayered CK3/12+ epithelium [210, 211].

### 5.15. Poly(vinyl alcohol)

Poly(vinyl alcohol) is a transparent hydrogel with good mechanical strength. Poly(vinyl alcohol) shows low cell affinity, but when incorporated with collagen type I it can support a fully stratified corneal epithelium* in vitro* [212], but to support* in vivo* epithelialization poly(vinyl alcohol)-collagen requires the assistance of HAM [213].

## 6. Carrier-Free Transplantation

Nishida et al. reported a temperature-responsive polymer, that is, poly(*N*-isopropylacrylamide) (PIPAAm), that could release intact, transplantable epithelial sheets that retain stem cells and epithelial cells [214]. The copolymer PIPAAm-PEG is at present commercialized as Mebiol gel and is hydrophilic at temperatures below 20°C and hydrophobic at temperatures above. Experiments have shown that Mebiol supports LESC cultivation* in vitro* and that autologous CLET in Mebiol restores the ocular epithelial surface in a LSCD rabbit model. The particular properties of Mebiol gel allow for easy graft transplantation. Drops of cooled Mebiol gel containing cultured LESCs can be applied to the ocular surface and a contact lens placed over it to keep it in place [215].

Furthermore,* in vitro* fibrin degradation, biodegradable type I collagen, and centrifugation proved to be effective techniques in fabricating carrier-free epithelial sheets. Cultured cells did proliferate and differentiate under the respective conditions, and cell-survival in the subsequent carrier-free state was preserved [216–218].

## 7. Alternative Cell Populations

LSCD frequently manifests as a bilateral condition where no residual stem cells are available for* ex vivo *culture. Allograft material from living related donors or cadavers may be used, but this is associated with an increased risk of disease transmission, rejection, and neoplasia (associated with immunosuppressive agents). Alternative cell populations could potentially replace the use of allogenic material and within the last decade a number of approaches have been explored with varying success [219].

### 7.1. Oral Mucosal Epithelial Cells

In 2003, Nakamura et al. described Cultivated Oral Mucosal Epithelial Transplantation (COMET) in a rabbit animal model [220]. Oral Mucosal Epithelial Cells (OMECs) are cultured on a HAM until a stratified epithelium is attained and then transplanted. The construct mimics the corneal epithelium as transplanted stem cells maintain their stemness at the ectopic site, and OMECs acquire corneal epithelial-like markers such as CK3, CK19, Ki-67, p63, p75, and cornea-specific PAX6 and CK12 [221–223]. COMET has been successful (i.e., regenerating a totally epithelized, stable, and avascular corneal surface) in patients with severe total LSCD [221, 223–232]. However, transplanted cultivated sheets are not completely identical to* in vivo* corneal epithelium, which leads to a variable degree of* in vivo* keratinization and stratification (up to 12 cell layers) [221, 228]. Small case series favour CLET, as COMET is associated with higher rates of peripheral corneal neovascularisation, inferior best corrected visual improvement, and increased risk of dry eye conditions postoperatively [221, 228].

### 7.2. Conjunctival Epithelial Cells

Human conjunctival epithelial cells grown on HAM have been used to reconstruct the ocular surface in rabbits with LSCD [233]. The transplanted conjunctival call sheets formed a five- to six-layer epithelium that remained transparent, smooth, avascular, and without epithelial defects [234]. Transplanted cells keep expressing both conjunctival (CK4) and corneal epithelial markers (CK3/12). Human conjunctival epithelial cell transplantation has been used clinically [235] and in one study in conjunction with a contact lens, which was removed at day 22 [43]. Almost 2 years after successful transplantation, a well-formed epithelium with 5 to 6 layers was present with rare PAS-positive cells, and positivity for CK3, CK19, P63, connexin 43, and MUC5AC [235]. Best corrected visual acuity significantly improved postoperatively, yet the effect was rather modest compared to CLET. Pain and photophobia were not being evaluated.

### 7.3. Hair Follicle Bulge-Derived Epithelial Stem Cells

Unlike OMECs, epithelial stem cells derived from the bulge region of the hair follicle are able to terminally differentiate into a corneal epithelial phenotype when transplanted onto the ocular surface [236]. The concept was proven in an animal study, in which hair follicle stem cells were cultured on a 3T3 feeder layer and transplanted into a LSCD mouse model [237]. The grafts were able to reconstruct the ocular surface in 80% of transplanted animals [237].

### 7.4. Amniotic Epithelial Cells

Human amniotic epithelial cells are characterized by their stem cell properties, low immunogenicity, production of growth factors that promote epithelialization, and their ability of controlled transdifferentiation into other cell types [238–241]. Amniotic epithelial cells can differentiate into corneal epithelial cells when seeded on the superficial corneal stroma in rabbit LSCD models [238–240, 242]. The differentiated cells had a similar structure, morphology, and physiology as that of normal stratified corneal epithelium. However, one study indicated that the stratified epithelial cells had no polarity with regard to defined superficial corneal epithelial cells, wing cells, or basal cells [238].

### 7.5. Human Embryonic Stem Cells

Human embryonic stem cells are pluripotent cells derived from the inner cell mass of the human embryo and can successfully differentiate into corneal epithelial-like cell [243, 244]. In a study from Zhu et al., human embryonic stem cells were induced to form LESC-like cells and were seeded on an acellular porcine corneal matrix [245]. Seeded cells formed stratified and closely arranged epithelioid cell sheets consisting of a basal layer of cuboid-shaped cells (p63a and ABCG2 positive) and suprabasal layers of elongated cells (CK3 positive). In rabbit LSCD models, the tissue engineered graft had the potential to reconstruct the ocular surface [245]. Embryonic stem cells also differentiate into corneal epithelial cells when in direct contact with the corneal stroma [246]. A major drawback to the use of human embryonic stem cells is the immune response they elicit, and the ethical controversy surrounding the origin of the stem cells [244, 247].

### 7.6. Induced Pluripotent Stem Cells

Induced Pluripotent Stem Cells (iPSCs) are a type of stem cells generated by manipulation of differentiated adult cells. In 2006, the iPSC technique was first described by Takahashi and Yamanaka and used four specific transcription factors to dedifferentiate adult cells into PSCs [248]. Hayashi et al.described a strategy to differentiate LESCs from human iPSCs that were derived from human adult corneal limbal epithelial cells or human dermal fibroblasts [249]. The iPSCs derived from adult corneal limbal epithelial cells gave rise to more corneal epithelial colonies and exhibited higher expression of specific corneal epithelial differentiation markers than iPSCs derived from fibroblasts [249, 250]. This may be due to the maintenance of epigenetic characteristics of the original adult cell during iPSC formation and subsequent differentiation [250, 251]. A significant drawback of the iPSC technique is that not all limbal epithelial cells preferentially differentiate into corneal epithelial cells [249]. Recently, a two-step differentiation method was developed to differentiate human iPSCs into a homogenous population of p63-positive epithelial cells with the ability to differentiate into corneal epithelial-like cells [252].

### 7.7. Umbilical Cord Lining Epithelial Stem Cells and Wharton's Jelly Mesenchymal Stem Cells

In 2011, Reza et al. described umbilical mucin-expressing cord lining epithelial stem cells as an alternative cell population in anterior corneal reconstruction [253]. These cells are nontumorigenic, highly proliferative, and ethically acceptable. The cells' low immunogenicity may obviate the postoperative use of immunosuppressants.* In vivo *verification in a rabbit model showed clear corneal surface regeneration with phenotypical CK3/CK12 expression [253]. Wharton's Jelly Mesenchymal Stem Cells have also been proposed for anterior corneal tissue engineering. Garzón et al. demonstrated that these MSCs could transdifferentiate* in vitro *into corneal epithelial-like cells, with the expression of epithelial cell markers (CK3/CK12, PKG, ZO1, and Cnx43) [254].

### 7.8. Mesenchymal Stem Cells

In 2006, Ma et al. were the first to expand MSCs on HAM and subsequently transplant the construct onto the ocular surface of LSCD rats [255]. Although bone marrow-derived human MSCs did not differentiate into epithelial-like cells, the transplanted MSCs successfully reconstructed the damaged corneal surface as a smooth and continuous epithelium, and avascular and transparent cornea were being observed [255]. The therapeutic effect may be due to the MSCs' anti-inflammatory and antiangiogenic properties, rather than direct epithelial differentiation. Gu et al. subsequently succeeded in differentiating rabbit-derived bone marrow MSCs into corneal epithelial-like cells [256].* In vitro, *differentiation was modulated by either (i) coculturing rabbit LESCs with MSCs or (ii) adding a LESC-derived supernatant to the MSCs [256]. Several other methods of inducing MSC differentiation have since been described [257–259]. In a LSCD rat model, corneal epithelial-like differentiation was modulated by cytokines, produced by rat Corneal Stromal Cells [257]. In 2011, Reinshagen et al. injected enriched MSCs under an AMT in LSCD rabbits [258]. Data indicated that injected MSCs may maintain their stem cell character or may differentiate into epithelial progenitor cells. More recently, it has been discovered that bone marrow-derived MSCs are capable of differentiating into corneal epithelial-like cells, when cultured in specialized DMEM-medium [259]. Adipose tissue-derived MSCs and limbal MSCs also can differentiate into corneal epithelial-like cells when exposed to (i) secreted factors of differentiated human corneal epithelial cells or (ii) DMEM-medium, respectively [260–263].

### 7.9. Human Immature Dental Pulp Stem Cells

Human immature dental pulp stem cells express both MSC and embryonic stem cell markers and have the capacity to differentiate into derivatives of the three germinal layers* in vitro*. In a LSCD rabbit experiment, transplanted human immature dental pulp stem cells were capable of reconstructing the ocular surface with a well-formed corneal epithelium that expresses LESC markers in the basal cell layer and EC markers in suprabasal cell layers [74, 264].

## 8. Conclusion

Over the past few years, great advances in LESC identification and characterization and ocular surface reconstruction have been made. With the introduction of CLET and SLET, a safe and successful treatment option for LSCD has been introduced [22–60, 94–97, 119, 120]. In particular, the tendency towards (i) standardized nonxenogenic GMP protocols in scaffold manufacturing and cell cultivation and (ii) “no touch graft surgery” is expected to improve success rates in future CLET trials [52, 55, 58, 59]. SLET seems to be very promising [94–97, 119, 120]; however, large cohort inclusion, allogenic transplantation, and long-term follow-up have yet to be performed. Further elaboration of “tear sampling” as a tool to identify factors that may be involved in the development and/or maintenance of corneal neovascularization in humans has been described [265]. This technique may assist in monitoring the inflammatory state of the LSCD eye and further improve preoperative management and postoperative outcome of patients. However, specific identification of the LESCs remains a hurdle and characterization is still based on a combination of phenotypic expression patterns [266]. Despite the successes and evolving techniques in LESC transplantation, detailed interaction and signaling pathways between LESCs, niche cells, and surrounding extracellular matrix are not fully understood. Research and knowledge within these domains will help understand (i) physiological LESC maintenance, (ii)* in vitro* and* in vivo* microenvironment simulation, and (iii) long-term effectiveness of LESC transplantation. Such knowledge may potentiate the development of new pharmacological solutions (e.g., eye drops that contain LESC growth factors) that stimulate remaining dormant LESCs of the diseased eye. These alternatives would be of great value in cases of extensive ocular inflammation, as these patients are not good candidates for surgical intervention.

Better* in vitro* and* in vivo* replication of the niche may also lead to more efficient cultivation and transplantation of LESCs and alternative cell populations. Of the investigated alternative seeding membranes, only HAM, fibrin, Siloxane Hydrogen contact lens, and collagen membranes have been used in patients [22–60, 94–97, 119, 120, 149, 151]. In particular, the conditional approval of Holoclar (Chiesi, Italy) is a huge step forward in the accessibility of LSCD treatment in daily practice. Furthermore, RHC membranes seem to be very promising for tissue engineering, the collagen being of nonxenogenic origin and the addition of MPC addressing many shortcomings of conventional collagen hydrogels. Other alternative scaffolds are still in an experimental phase and have yet to be validated in humans. COMET and human conjunctival epithelial cell transplantation have both been successfully performed in selected patients [43, 221, 223–232, 235]. However, as iPSCs get widespread attention in many medical disciplines, it is believed that this autologous cell population will play a prominent role in LSCD treatment in the coming years.

In conclusion, it can be certain that better and more convenient treatment options for LSCD patients will emerge in the near future. New treatment options will target optical transparency, biocompatibility, intraoperative handling, physicochemical strength, and cost-effectiveness. The important focus on sterility, reproducibility, and minimal mutagenicity and cytotoxicity is further stimulated by the widespread introduction of GMP guidelines.

## Figures and Tables

**Table 1:** Aetiology of LSCD.

Primary causes	Reference
Aniridia	[67, 71, 72]
Multiple endocrine deficiency	[9, 67]
Epidermal dysplasia	
Ectrodactyly-ectodermal-dysplasia-clefting syndrome	[73]
Congenital erythrokeratodermia	[74]
Dyskeratosis congenita	[75, 76]

Secondary causes	

Thermal or chemical burns	[67, 77]
Contact lens wear	[67, 78]
Inflammatory eye disease:	
Stevens-Johnson syndrome, toxic epidermal necrolysis	[67]
Ocular cicatricial pemphigoid	[79]
Chronic limbitis: autoimmune disease, extensive microbiological infection, atopic conjunctivitis	[80]
Neurotrophic keratitis	[80]
Extensive limbal cryotherapy, radiation, or surgery	[81]
Bullous keratopathy	[82]
Topical antimetabolites (5-FU, Mitomycin C)	[83, 84]
Systemic chemotherapy (Hydroxyurea)	[85]

5-FU: 5-fluorouracil.

**Table 2:** Treatment options for limbal stem cell deficiency.

Procedure	Mechanism of action and remarks	References
*Conservative nonsurgical options*		

Autologous serum drops	Serum drops promote migration and proliferation of healthy epithelium while lubricating the ocular surface, preventing epithelial adhesion to the tarsal conjunctiva, and reducing shear stress.	[86–88]

Therapeutic soft contact lens	Therapeutic lenses promote healing of persistent epithelial defects (PED) and prevent the formation of new defects.	[89]

Therapeutic scleral lens	Scleral lenses promote healing of PED while improving vision (optical effect) and reducing pain and photophobia (therapeutic effect). They also prevent formation of new epithelial defects.	[90]

Eye lubrication	Ocular surface lubrication prevents epithelial adhesion to the tarsal conjunctiva and reduces shear stress. Unlike autologous serum drops, residual stem cell migration and proliferation is not enhanced.	[89]

*Conservative surgical options*		

Corneal scraping	During scraping the overgrown conjunctiva is removed, enabling reepithelialisation by islands of functioning corneal epithelial stem cells. However, because the conjunctival epithelium migrates more rapidly than the corneal epithelium, it may be necessary to repeat the procedure two to three times.	[91]

Amniotic membrane transplantation (AMT)	AMT promotes proliferation and migration of residual LESCs, contributing to the recovery of the corneal surface, improved visual acuity, and alleviation of pain and photophobia. Low immunogenicity, and anti-inflammatory, antiangiogenic, antifibrotic, antimicrobial, and antiapoptotic properties of the amniotic membrane assist in its therapeutic effect. An AMT is performed immediately after corneal scraping as the overgrown conjunctiva is removed and the amnion membrane is patched over the epithelial defect. Variable clinical outcome may be attributed to inter- and intradonor variation of the biologically sourced membrane.	[88, 92]

*Limbal epithelial stem cell transplantation*		

Conjunctival limbal autograft (CLAU)	Autologous graft derived from the patient's healthy eye, using the conjunctiva as carrier tissue. As this procedure involves dissecting 2 clock hours each of limbal tissue superiorly and inferiorly, CLAU holds the risk of inducing LSCD in the healthy donor eye.	[6, 21, 50, 89]

Conjunctival limbal allograft (CLAL)	Allogenic graft derived from a living related (lr-CLAL) or deceased donor (c-CLAL), using the conjunctiva as carrier tissue. CLAL comes with an increased risk of transmitting infectious disease and promoting neoplasia due to the long-term use of immunosuppressants. The surgical procedure and number of clock hours to be dissected are similar to that for CLAU. Lr-CLAL may induce LSCD in the healthy donor eye.	[6, 21, 50, 89]

Keratolimbal allograft (KLAL)	Allogenic graft derived from a deceased donor, using the cornea as carrier tissue. As in CLAL, there is an increased risk of disease transmission and formation of neoplasia. KLAL requires approximately 6 clock hours of tissue to be removed from the donor limbus and transplanted onto the stem cell deficient eye.	[6, 21, 50, 89]

*Ex vivo* cultivated limbal epithelial stem cells (CLET)	Autologous or allogenic transplantation of cultivated stem cells, most commonly using the human amniotic membrane or fibrin as a carrier for the composite graft. The major advantage of this technique is the reduced risk of inducing LSCD in the healthy donor eye, and the decreased incidence of immunological rejection as Langerhans cells are not cultured in the composite graft. However, the use of HAM or the transplantation of allogenic LESCs bears the risk of disease transmission. Furthermore, the use of immunosuppressants may be necessary in allogenic transplantation with limited HLA-compatibility. Finally, some culture protocols use animal-derived products, which pose the theoretical risk of zoonosis and/or elicit an immune response in the acceptor.	[21, 22, 59, 93]

Simple limbal epithelial transplantation (SLET)	Autologous transplantation of tiny limbal grafts that are distributed and glued evenly over a HAM. Circumventing difficulties of *ex vivo* culture techniques, epithelialisation is achieved *in vivo*. As seen in CLET, there is limited risk of immunological rejection or induction of LSCD in the healthy donor eye. Furthermore difficulties of e*x vivo* culturing are avoided, promoting cost-effectiveness. However, the rate of LESC expansion *in vivo* must be greater than that of the rapidly proliferative conjunctiva to attain successful engraftment.	[94]

**Table 3:** Culture methods and clinical results of published CLET reports.

	Patients	Type of graft	Substrate	3T3s used	Animal Free culturing conditions	GMP	Success rate	2-line visual improvement	Subsequent surgery	Complications	Follow-up (months)	
											Mean	Range
Ang et al. [38]	1	Allograft	HAM (denuded)	+	+	−	100% (1/1)	0% (0/1)	—	—	48	—

Baradaran-Rafii et al. [45]	8	Autograft	HAM (denuded)	−	−	−	88% (7/8)	63% (7/8)	KP (4)	Perforation (1)	34	6–48

Basu et al. [55]	50	Autograft	HAM	−	+	−	66% (33/50)	76% (38/50)	KP (8)	Bleeding (23), bacterial keratitis (1)	28	12–90

Daya et al. [34]	10	Allograft	3T3s	+	−	−	70% (7/10)	33% (3/9)	KP (5), cataract (1), KLAL (5)	Infective keratitis (1)	28	12–50

Di Girolamo et al. [43]	2	Autograft	Siloxane Hydrogel CL	−	−	−	100% (2/2)	50% (1/2)	—	—	10.5	8–13

Di Iorio et al. [48]	166	Autograft	Fibrin	+	−	−	80% (133/166)	—	KP (33)	—	—	>6

Fatima et al. [41]	1	Autograft	HAM	−	−	−	100% (1/1)	100% (1/1)	KP (1)	—	37	—

Gisoldi et al. [46]	6	Autograft	Fibrin	+	−	+	83% (5/6)	83% (5/6)	KP (4), cataract (1)	—	24	11–34

Grueterich et al. [29]	1	Autograft	HAM	−	−	−	100% (1/1)	100% (1/1)	KP (1), cataract (1)	—	21	—

Kawashima et al. [40]	6	Autograft (2), allograft (4)	HAM (denuded)	+	+	−	100% (6/6)	67% (4/6)	KP (6), cataract (5)	CRVO (1)	32	20–44

Koizumi et al. [26]	13	Allograft	HAM (denuded)	+	+	−	77% (10/13)	38% (5/13)	—	Rejection (3), infection (1), conjunctival invasion (2), conjunctival fibrosis (1)	11	6–13

Koizumi et al. [27]	3	Allograft	HAM (denuded)	+	+	−	100% (3/3)	0% (0/2)	—	—	6	—

Kolli et al. [50]	8	Autograft	HAM	−	−	+	100% (8/8)	63% (5/8)	KP (1), graft redo (1)	—	19	12–30

Meller et al. [44]	1	Allograft	HAM	−	−	−	100% (1/1)	100% (1/1)	—	Perforation (1)	31	—

Nakamura et al. [32]	3	Allograft	HAM (denuded)	+	+	−	100% (3/3)	33% (1/3)	—	—	13	12–14

Nakamura et al. [33]	1	Autograft	HAM (denuded)	+	+	−	100% (1/1)	100% (1/1)	—	—	19	—

Nakamura et al. [36]	9	Autograft (2), allograft (7)	HAM (denuded)	+	+	−	100% (9/9)	67% (6/9)	—	Infective keratitis (1)	14.6	6–20

Pathak et al. [58]	9	Autograft	HAM	−	+	−	56% (5/9)	33% (3/9)	KP (1), graft redo (1), AMT (1)	—	18.5	11–24

Pauklin et al. [51]	44	Autograft (30), allograft (14)	HAM	−	−	−	68% (30/44)	73% (32/44)	KP (8), cataract (5)	Bleeding (1), perforation (2)	28.5	9–72

Pellegrini et al. [22]	2	Autograft	3T3s	+	−	−	100% (2/2)	50% (1/2)	KP (1)	—	—	>24

Prabhasawat et al. [54]	19	Autograft (12), allograft (7)	Ham (denuded)	−	+	−	73.7% (14/19)	68.4% (13/19)	KP (6), lid correction (3), cataract (3), tarsorrhaphy (1)	Infection (3), PED (3), symblepharon (1)	26.1	6–47

Rama et al. [28]	18	Autograft	Fibrin	+	−	−	78% (14/18)	33% (6/18)	KP (3)	Persistent inflammation with bleeding (4)	17.5	12–72

Rama et al. [49]	107	Autograft	Fibrin	+	−	+	68% (73/107)	54% (61/107)	KP (62), PTK (2)	Bleeding (12), inflammation (59), herpetic keratitis (3), blepharitis/epitheliopathy (35), residual fibrin (11)	35	12–120

Sangwan et al. [31]	2	Autograft	HAM	−	−	−	100% (2/2)	50% (1/2)	—	Recurrence (1)	12	—

Sangwan et al. [31]	15	Autograft (11), allograft (4)	HAM	−	−	−	100% (15/15)	87% (13/15)	KP (15)	Rejection (4), glaucoma (1)	15.3	7–24

Sangwan et al. [37]	78	Autograft	HAM	−	−	−	73% (57/78)	37% (18/49)	KP (19)	Phthisis (2), keratitis (2), glaucoma (2)	18.3	3–40

Sangwan et al. [52]	200	Autograft	Ham (denuded)	−	+	−	71% (142/200)	60.5% (121/200)	—	Bleeding (56), PED (13), corneal melting (5), bacterial keratitis (3)	36	12–91

Schwab [23]	19	Autograft (17), allograft (2)	HAM	+	−	−	74% (14/19)	16% (3/19)	Graft redo (1)	—	10.5	2–24

Schwab et al. [24]	14	Autograft (10), allograft (4)	HAM (denuded)	+	+	−	71% (10/14)	36% (5/14)	KP (1)	Epithelial loss (1), cyclosporine-related (2), infectious keratitis (1), pyogenic granuloma (1)	13	6–19

Sejpal et al. [57]	107	Autograft	HAM (denuded)	−	+	−	49.5% (53/107)	54.2% (58/107)	KP (19), lid or fornix correction (16)	Infection (7), inflammatory granuloma (4), glaucoma (1), corneal thinning (1), bleeding (1), panophthalmitis (1)	41.2	12–118

Sharma et al. [53]	50	Autograft (34), allograft (16)	HAM (denuded)	−	−	−	74% (37/50)	68% (34/50)	KP (4)	—	11	1.5–25

Shimazaki et al. [30]	13	Allograft	HAM (denuded)	−	−	−	38.5% (5/13)	76.9% (10/13)	—	Perforation (4), infection (2)	—	—

Shimazaki et al. [39]	27	Autograft (7), allograft (20)	HAM (denuded)	+	−	−	59% (16/27)	48% (13/27)	KP (8), limbal transplant (3)	Infection (1), ulceration (4), perforation (4)	29.3	6–85

Shortt et al. [42]	16	Autograft (9), allograft (7)	HAM (denuded)	−	−	+	75% (12/16)	22% (2/9)	Graft redo (1)	Infection (1), cyclosporin related (1), graft detachment (1)	9.3	6–13

Subramaniam et al. [56]	40	Autograft	HAM (denuded)	−	−	−	45% (18/40)	—	KP (10)	—	33.4	1–87

Thanos et al. [47]	1	Autologous	HAM	−	−	−	100% (1/1)	100% (1/1)	—	—	24	—

Tsai et al. [25]	6	Autograft (3), allograft (3)	HAM (denuded)	−	−	−	100% (6/6)	50% (3/6)	—	—	15	12–18

Vazirani et al. [60]	70	Autograft	HAM (denuded)	−	−	−	71% (49/70)	—	—	—	17.5	—

Zakaria et al. [59]	18	Autograft (15), allograft (3)	HAM (denuded)	−	+	+	67% (12/18)	28% (5/18)	KP (7)	—	24	4–48

Overall	1164	Autograft (1029), allograft (135)					70.26%	54.92%			25.4	1–120

GMP: good manufacturing practice; HAM: human amniotic membrane; CL: contact lens; KP: keratoplasty; AMT: amnion membrane transplantation; PTK: phototherapeutic keratectomy; CRVO: central retinal vein occlusion; PED: persistent epithelial defect.

**Table 4:** Published clinical outcomes of SLET.

	Patients	Type of graft	Substrate	Success rate	2-line visual improvement	Subsequent surgery	Complications	Follow-up (months)	
								Mean	Range
Amescua et al. [95]	4	Autograft	HAM	100% (4/4)	100% (4/4)	—	—	7.5	6–9
Bhalekar et al. [96]	1	Allograft	HAM	100% (1/1)	100% (1/1)	—	Rejection	6	—
Bhalekar et al. [119]	1	Autograft	HAM	100% (1/1)	100% (1/1)	—	—	>1	—
Bhalekar et al. [120]	1	Autograft	HAM	100% (1/1	100% (1/1)	—	Epithelial plaque hyperplasia	14	—
Vazirani et al. [97]	1	Autograft	HAM	100% (1/1)	100% (1/1)	Graft redo, conjunctival autografting	—	6	—
Sangwan et al. [94]	6	Autograft	HAM	100% (6/6)	100% (6/6)	—	—	9.2	4–48

Overall	14			100%	100%			8	4–48

HAM: human amniotic membrane.
